# Vitellogenin receptor transports the 30K protein LP1 without cell-penetrating peptide, into the oocytes of the silkworm, *Bombyx mori*

**DOI:** 10.3389/fphys.2023.1117505

**Published:** 2023-01-26

**Authors:** Yinying Xu, Guanwang Shen, Jinxin Wu, Xueqin Mao, Linbang Jia, Yan Zhang, Qingyou Xia, Ying Lin

**Affiliations:** ^1^ State Key Laboratory of Silkworm Genome Biology, Southwest University, Chongqing, China; ^2^ Biological Science Research Center Southwest University, Chongqing, China; ^3^ Integrative Science Center of Germplasm Creation in Western China (Chongqing) Science City & Southwest University, Chongqing, China; ^4^ Chongqing Key Laboratory of Sericultural Science, Chongqing, China

**Keywords:** vitellogenin receptor, lipoprotein 1, 30k proteins, transport, *Bombyx mori*

## Abstract

Vitellogenin receptors (VgRs) transport vitellogenin (Vg) into oocytes, thereby promoting egg growth and embryonic development. VgRs recognize and transport multiple ligands in oviparous animals, but their role in insects is rarely reported. In this study, we investigated whether *Bombyx mori* VgR (BmVgR) binds and transports lipoprotein-1 (BmLP1) and lipoprotein-7 (BmLP7) of the 30 kDa lipoproteins (30 K proteins), which are essential for egg formation and embryonic development in *B. mori*. Protein sequence analysis showed BmLP7, similar to reported lipoprotein-3 (BmLP3), contains the cell-penetrating peptides and Cysteine position, while BmLP1 has not. Assays using *Spodoptera frugiperda* ovary cells (sf9) indicated the direct entry of BmLP7 into the cells, whereas BmLP1 failed to enter. However, co-immunoprecipitation (Co-IP) assays indicated that BmVgR could bind BmLP1. Western blotting and immunofluorescence assays further revealed that over-expressed BmVgR could transport BmLP1 into sf9 cells. Co-IP assays showed that SE11C (comprising LBD1+EGF1+OTC domains of BmVgR) or SE22C (comprising LBD2+EGF2+OTC domains of BmVgR) could bind BmLP1. Over-expressed SE11C or SE22C could also transport BmLP1 into sf9 cells. Western blotting revealed that the ability of SE11C to transport BmLP1 might be stronger than that of SE22C. In the *vit* mutant with *BmVgR* gene mutation (*vit/vit*), SDS-PAGE and western blotting showed the content of BmLP1 in the ovary, like BmVg, was lower than that in the normal silkworm. When transgenic with hsp70 promoter over-expressed BmVgR in the *vit* mutant, we found that the phenotype of the *vit* mutant was partly rescued after heat treatment. And contents of BmLP1 and BmVg in *vit* mutant over-expressed BmVgR were higher than in the *vit* mutant. We conclude that BmVgR and its two repeat domains could bind and transport BmLP1 into the oocytes of the silkworm, besides BmVg. These results will provide a reference for studying the molecular mechanism of VgR transporting ligands in insects.

## Introduction

In insects, vitellogenin receptors (VgRs) belong to the low-density lipoprotein receptor (LDLR) family, which is able to transport Vg into the oocytes and provide nutrition for eggs growth and embryonic development (Sappington and Raikhel, 1998). VgR is a crucial receptor for Vg transport and a potential target for pest control (Snigirevskaya et al., 1997; Lian et al., 2022). Studies have shown that VgRs can also recognize and transport multiple ligands. For example, the VgR in chickens recognizes at least eight different ligand molecules (Jacobsen et al., 1995). Low-density lipoprotein receptor-related protein (LRP) is known to bind to more than 20 different ligands (Strickland et al., 1995; Kounnas et al., 1996). However, fewer studies have focused on establishing the role of VgRs, including those of BmVgR, on whether they could transport multiple ligands other than Vg in insects.

The silkworm *vit* mutant lacks vitellin (BmVn) and 30 K proteins, although their precursors are abundant in the pupal hemolymph (Fujikawa et al., 1995). The *vit* eggs appear whiter and smaller than wild-type (WT) and homozygous lethal eggs (Lin et al., 2013). Lin et al. attributed the phenotype of *vit* to the mutated BmVgR with a deletion in the third-class B region of the epidermal growth factor 1 (EGF1) domain. This led to the retention of its ligand-binding ability but hampered its ligand dissociation under acidic conditions. Thus, BmVgR was unable to return to the cell membrane to transport other ligands. Eventually, this led to the lack of BmVn and 30 K proteins in the eggs of the *vit* mutant (Lin et al., 2013). These studies suggest that BmVgR may be responsible for transporting 30 K proteins in silkworm.

The 30 K proteins family of silkworm are a group of structurally related proteins of molecular weights approximately 30 kDa; they comprise 46 members, which are classified under three subfamilies: typical 30 kDa lipoproteins (BmLP1–24), serine/threonine-rich lipoproteins (BmLP25–36), and ENF peptide-binding proteins (BmLP37–46) (Zhang et al., 2012a). 30 K proteins and BmVg are abundant in silkworm hemolymph (Mori et al., 1991). In freshly laid silkworm eggs, Vg accounts for 40% and 30 K proteins account for 35% of the total egg protein (Qian, 1995). Similar to BmVg, the 30 K proteins are synthesized by the fat body and are secreted into the hemolymph during the fifth-instar larval to the early pupal stages of silkworms (Bosquet et al., 1989; Mori et al., 1991). From the first day of pupation to the moth stage, they gradually transfer from hemolymph to oocytes (Zhong et al., 2005). The 30 K proteins predominantly function as storage proteins to provide energy for embryonic development and the hatching of silkworms. However, their transport mechanism is still unclear. The 30 K proteins of silkworms are a group of proteins that are highly similar in nucleotide and amino acid sequences (Mori et al., 1991). Park et al. found that BmLP3 (30Kc19), one of the 30 K proteins, had cell-penetrating properties and revealed that it entered cells by forming a dimer at its Cysteine position, followed by macropinocytosis and caveolin-mediated endocytosis (Park et al., 2014b). Furthermore, they reported that a cell-penetrating peptide (CPP) derived from BmLP3 protein, VVNKLIRNNKMNC, including the Cysteine position, could efficiently penetrate cells grown in a medium used for mammalian cell culture (Park et al., 2014a). This is an indication that some of the 30 K proteins without CPP may need receptors to be transported into the oocytes of silkworms.

In addition to BmVg, BmVgR might also transport BmLP1, one of the 30 K proteins without CPP. In this study, we downloaded the 30 K protein sequences and analyzed their structure. Co-IP assay was performed to reveal whether BmVgR could bind to BmLP1 and whether the two ligand-binding domains (LBRs) of BmVgR were involved in the transport of BmLP1. Cell incubation assays were performed to determine whether BmVgR could transport BmLP1 into insect cells by pHrodo-Red labeling. Studies using the *vit* mutant and transgenic *vit* mutant were used to identify whether BmVgR could transport BmLP1 into silkworm oocytes.

## Materials and methods

### Insects and tissue collection

The silkworm *vit* mutant heterozygote *vit/+* strain (*vit oh/+ +*♀ × *vit oh/vit oh*♂) used in this study was obtained from Kyushu University (Silkworm Base). The *vit* mutant *vit/vit* (*vit oh/vit oh*, *BmVgR* mutant homozygote) and *dazao* (wild type) strains were maintained at Southwest University, China. The silkworm transgenic *BmVgR + vit* (*BmVgR* overexpressed in *vit* mutant) strain was generated in our laboratory by repeatedly mating the transgenic *BmVgR* strain with the *vit* mutant. The transgenic *BmVgR* strain was developed using transgenic *BmVgR/BmVgR-*positive individuals selected by injecting the transgenic plasmid *piggyBac-[3×p3-EGFP-SV40]-[hsp70-BmVgR-SV40]* into silkworm eggs. The Hsp70 promoter used in the construct was that of the *Drosophila melanogaster* heat-shock protein 70 gene. All larvae were reared on fresh mulberry leaves; pupae and moths were maintained at 25°C, 75% ± 5% relative humidity, and a photoperiod of 12 h light/12 h dark. All hemolymph samples were collected into ice-cooled tubes containing a few of phenylthiourea crystals and 1 mM phenyl methyl sulfonyl fluoride (PMSF). After haemocytes were removed by centrifugation at 6,000 *g* at 4°C for 20 min, and stored at −80°C. Ovaries were dissected from female pupae and moths, washed using physiological saline with diethyl pyrocarbonate, and stored at −80°C.

### Protein preparation and detection

Protein samples were obtained from the ovaries at different phases in the silkworm pupae or from sf9 cells (*Spodoptera frugiperda* ovary cells). The silkworm ovaries from each phase were homogenized using liquid nitrogen. Tissue and cellular total proteins were extracted using a kit as per the manufacturer’s instructions (Beyotime, Shanghai, China). NP40 lysate buffer (Beyotime) (containing 1 mM PMSF) was added to the powder or the cells, and the mixture was lysed on ice for 30 min. This was followed by centrifugation at 12,000 *g* for 10 min, and the resultant supernatant was collected.

The supernatant was subjected to sodium dodecyl-sulfate polyacrylamide gel electrophoresis (SDS–PAGE; 10% w/v polyacrylamide gel) as described by Laemmli (Laemmli, 1970). The gel was stained using Coomassie brilliant blue (Wako, Osaka, Japan). For western blotting analysis, proteins (30 μg) in the gel were transferred electrophoretically onto a polyvinylidene fluoride membrane (Roche, Basel, Switzerland). The membrane was blocked with 5% (w/v) dry skim milk for 2 h and then incubated with the antibody at a dilution of 1:10,000 with Tris-buffered saline containing Tween 20 (TBST) for 2 h and then washed five times, 7 min each with TBST. The membrane was then treated for 1 h with horseradish peroxidase (HRP)-labeled goat anti-rabbit/mouse IgG, used as the secondary antibody (diluted 1:10,000 with TBST), followed by washing with TBST five times, each for 7 min. Signals were detected using the Super Signal^®^ West Pico Chemiluminescent Substrate (Thermo Fisher Scientific, Waltham, MA, United States) under a ChemiScope Imaging System.

### BmLP1 and BmLP7 proteins purification

The BmLP1 and BmLP7 proteins were purified from silkworm *dazao* hemolymph as previously described (Luo et al., 2014). On day two of pupation, the hemolymph was diluted in 20 mM Tris–HCl and 100 mM NaCl buffer (pH 7.5) and centrifuged at 6,000 × *g* at 4°C for 20 min. The highest amount of 30 K proteins was detected in 25%–50% ammonium sulfate saturation; the sample was dialyzed against TBS buffer (containing 1 mM PMSF) at 4°C. The dialyzed sample was then purified using Q column (HiTrapQ HP anion exchange column). After verification using SDS-PAGE, the desired proteins were gathered and applied to a HiLoad Superdex S-75 16/600 column purification (GE Healthcare, United States). The desired proteins were collected. The purified proteins were further confirmed using SDS-PAGE. The recombinant BmLP1 protein (His-BmLP1) was generated in our laboratory (Ye et al., 2021).

### Cell expression vector construction and expression

The 1180 (Hrs1000-BmActin4-SV40) expression vector was maintained in our laboratory. We obtained full-length BmVgR from p50 T6kb-9 plasmid DNA (offered by Prof Yan Meng at Anhui Agricultural University in China), 1180-BmVgR (Hrs1000-BmActin4-SP-LBD1-EGF1-LBD2-EGF2-OTC-Ser1PA), 1180-S11C (Hrs1000-BmActin4-SP-LBD1-EGF1-OTC-Ser1PA), and 1180-SP-EGFP-C (Hrs1000-BmActin4-SP-EGFP-C-Ser1PA) (stored at our laboratory).

Before the SP-EGFP-LBD1-EGF1-OTC (SE11C) was obtained, we performed polymerase chain reaction (PCR) amplification of SP-EGFP and LBD1-EGF1-OTC. The primers used for PCR are listed in Table 1. The SP-EGFP fragment was PCR-amplified using pfu Taq DNA polymerase (Promega, United States), with SP-EGFP-F and SP-EGFP-R primers (Table 1); 1180-SP-EGFP-C was used as the template. The PCR conditions were 94°C for 5 min, followed by 35 cycles of 94°C for 30 s, 50°C for 30 s, and 72°C for 50 s, with a final extension at 72°C for 10 min. The LBD1-EGF1-OTC fragment was PCR-amplified using PrimerSTAR Max DNA polymerase (Takara Bio, Otsu, Japan); the primers were LBD1-EGF1-OTC-F and C-R (Table 1); 1180-S11C was used as the template. The PCR conditions were 94°C for 5 min; for each cycle, the annealing temperature was increased by one degree, with a total of ten cycles of 94°C for 30 s, 45–54°C for 30 s, and 72°C for 30 s; 30 cycles of 94°C for 30 s, 55°C for 30 s, and 72°C for 30 s; and a final extension at 72°C for 10 min. Next, SP-EGFP and LBD1-EGF1-OTC were used as templates, and SE11C was amplified using homologous recombination PCR. The PCR primers were SP-EGFP-F and C-R (Table 1); the resulting cDNA fragment was PCR-amplified using LA-Taq DNA polymerase (Takara Bio, Otsu, Japan). The PCR conditions were 94°C for 5 min, followed by 35 cycles of 94°C for 30 s, 50°C for 30 s, and 72°C for 3 min, and a final extension at 72°C for 10 min. The amplification process of SP-EGFP-LBD2-EGF2-OTC (SE22C) was the same as that of SE11C. To obtain SE22C, we performed PCR amplification of SP-EGFP’ and LBD2-EGF2-OTC. SP-EGFP’ was amplified using SP-EGFP-F and SP-EGFP’-R primers (Table 1). The PCR conditions and templates were the same as those for SP-EGFP. LBD2-EGF2-OTC was amplified using LBD2-EGF2-OTC-F and C-R primers (Table 1). 1180-BmVgR was used as a template. The PCR conditions were the same as those for LBD1-EGF1-OTC. SE22C was amplified using SP-EGFP-F and C-R primers (Table 1) *via* homologous recombination PCR; SP-EGFP’ and LBD2-EGF2-OTC were used as templates. The PCR conditions were 94°C for 5 min, followed by 35 cycles of 94°C for 30 s, 50°C for 30 s, and 72°C for 2 min 40 s, and a final extension at 72°C for 10 min. All PCR products were confirmed by electrophoresis on a 1.2% (w/v) agarose gel and photographed using the Molecular Imager Gel Doc XR system (Bio-Rad, United States).

**TABLE 1:** Primer for this study.

Primer name	Sequence
SP-EGFP-F	5ʹ-TGT​TAG​AGG​ATT​GGT​GGA​TCC​ATG​AAG​GTA​GTT​TTG​TTA​GCA​ATA​G-3ʹ ʹ
SP-EGFP-R	5ʹ-ACG​GAA​ACA​CAT​CCT​CGC​CCA​GGC​AAG​AGC​CTC​CAC​CCC​CCT​TGT​ACA​GC-3ʹ
LBD1-EGF1-OTC-F	5ʹ-GCT​GTA​CAA​GGG​GGG​TGG​AGG​CTT​GCC​TGG​GCG​AGG​ATG​TGT​TTC​CGT-3ʹ
C-R	5ʹ-TCG​TGT​TTA​GTT​GTA​GCG​GCC​GCT​AAT​TGA​GAA​ATT​TAT​TTT​TCC​GGA​TG-3ʹ
SP-EGFP’-R	5ʹ-GAC​AGT​AAA​TTT​CGC​TTT​CGC​TGC​AAG​AGC​CTC​CAC​CCC​CCT​TGT​ACA​GC-3ʹ
LBD2-EGF2-OTC-F	5ʹ-GCT​GTA​CAA​GGG​GGG​TGG​AGG​CTC​TTG​CAG​CGA​AAG​CGA​AAT​TTA​CTG-3ʹ

ATG​AAG​GTA​GTT​TTG​TTA​GCA​ATA​G, is belong to the signal peptide (SP) of BmVgR, GCTGTACAAG, is belong to EGFP, fragment, GGGGGTGGAGGCTCT, is linker fragment, other sequences of primers are belong to CDS, of BmVgR.

After the amplified fragments were confirmed by sequencing, the fragments were ligated into the 1180 expression vector using the Ligation-free Cloning System (Abm, Vancouver, Canada). The vectors were named SE11C and SE22C and were confirmed by sequencing. The sf9 cell line was used for cellular assays since the endogenous genes of BmVgR, BmLP1, and BmLP7 are not expressed in sf9 cells. The cells were transfected with SE11C or SE22C plasmid DNA using Cellfection II kit (Invitrogen Carlsbad, California, United States), following the manufacturer’s instructions. After the complete medium was replaced with fresh medium and the cells were cultured for 72 h, total protein from the cells was extracted using NP40 lysis buffer (Beyotime); the expression of SE11C or SE22C protein was analyzed using western blotting with anti-GFP polyclonal rabbit antibody (1:10,000) (Genscript, New Jersey, United States) and HRP-conjugated goat anti-rabbit IgG (H + L) antibody (1:10,000) (Beyotime). The transfected cells were also used to determine the localization of existing SE11C or SE22C using a fluorescence assay. Detection of SE11C and SE22C in sf9 cells using green fluorescence under a laser confocal microscope (FV1000; Olympus, Tokyo, Japan).

### Co-IP assay

BmVgR protein was detected in the total protein samples of the ovaries of the silkworm *dazao* on day one of pupation using western blotting with anti-LBD2 polyclonal rabbit antibody (1:10,000). Recombinant antigen LBD2 (amino acid positions of BmVgR 934–1282) was expressed using the pET28a-BmVgR-LBD2 plasmid in *Escherichia coli* strain BL21 (DE3) (TransGen Biotech, France). SE11C/SE22C protein was obtained by expressing SE11C/SE22C in sf9 cells and detected using western blotting with anti-GFP polyclonal rabbit antibody (1:10,000). His-BmLP1 protein was added to the culture medium and incubated with the BmVgR/SE11C/SE22C protein at 4°C for 6 h. Approximately 7.5 μg of anti-His mouse monoclonal antibody (Genscript, New Jersey, United States) was then diluted in 200 μL of NP40 and added to 50 μL of 5% (w/v) BSA-blocked Dynabeads (Thermo Fisher Scientific). The Dynabeads were used for the experiment according to manufacturer’s instructions. The incubation medium was collected as a positive control. Interference of non-specific proteins was excluded by washing the membrane twice with phosphate-buffered saline (PBS; pH 6.5) and then once with PBS (pH 7.4), and the final wash buffer was used as a negative control. The complex was resuspended in 100 μL of PBS (pH 7.4) and transferred to a clean tube. Approximately 20 μL of 5 × SDS–PAGE loading buffer was then added, and the mixture was heated for 10 min at 100 °C. The eluted protein from the beads was subjected to SDS–PAGE (10% w/v polyacrylamide gel) and detected using western blotting with anti-LBD1 against recombinant antigen LBD1 (amino acid positions of BmVgR 27–224) expressed using pET28a-BmVgR-LBD1 in *Escherichia coli* strain BL21 (DE3) (TransGen Biotech, France) or anti-LBD2 rabbit antibody (1:10,000; anti-LBD1 and anti-LBD2 rabbit antibodies were prepared in our laboratory).

### Fluorescent labeling of BmLP1 and BmLP7 proteins

Fluorescent labeling of BmLP1 and BmLP7 proteins was performed using pHrodo-Red dye (Thermo Fisher Scientific), following the manufacturer’s instructions. The stock solution of the dye was prepared by dissolving 1 mg pHrodo-Red dye into 150 μl of dimethyl sulfoxide. The buffer for BmLP1 and BmLP7 proteins were replaced with freshly prepared 0.1 M NaHCO_3_ buffer (pH 8.3), and the proteins were diluted to 1 mg/mL prior to fluorescent labeling. An appropriate amount of reactive dye (dye 10: protein 1) was added to the protein solution in sodium bicarbonate buffer and mixed. The reaction was incubated for 1 h at 25 °C in the dark. The labeled BmLP1 and BmLP7 proteins were purified using ultrafiltration in the dark. The pHrodo-Red-labeled BmLP1 and BmLP7 proteins (BmLP1-Red and BmLP7-Red) prepared were stored at −80 °C until further use.

### Detection of BmLP1 transport by BmVgR into sf9 cells

The mechanism of entry of BmLP1 into sf9 cells was studied using 12-well cell culture plates (for western blotting) and 24-well cell culture plates (for immunofluorescence); BmLP7 was used as the control. Sf9 cells were starved for 30 min in Grace’s medium without fetal bovine serum and incubated with 200 μg/mL BmLP1-Red or BmLP7-Red protein for 60 min at 27°C. The cells were washed twice with PBS (pH 6.5) and then once with PBS (pH 7.4) to remove ligand proteins that failed to enter the cells. BmLP1 and BmLP7 proteins were detected using western blotting analysis with anti-BmLP1 and anti-BmLP7 rabbit antibodies (1:10,000) (anti-BmLP1 and anti-BmLP7 rabbit antibodies were prepared in our laboratory) (Zhang et al., 2012b), respectively, followed by HRP-conjugated goat anti-rabbit IgG (H + L) antibody (1:10,000). Fluorescence signals of cells were observed under a fluorescence microscope (Life Technologies, United States). Each experiment was performed three times.

To determine whether BmLP1 is transported by BmVgR, SE11C, or SE22C, each of these three proteins was overexpressed in sf9 cells and then analyzed using the methods described above. BmVgR and BmLP1 proteins were detected using western blotting with anti-LBD2 rabbit antibody (1:10,000) or anti-BmLP1 rabbit antibody (1:10,000), followed by HRP-conjugated goat anti-rabbit IgG (H + L) antibody (1:10,000). And the cells detected using immunofluorescence assay with anti-LBD2 rabbit antibody and goat anti-rabbit IgG (H + L) cross-adsorbed secondary antibody (1:10,000), Alexa Fluor™ 488 (Thermo Fisher Scientific); anti-BmLP1 rabbit antibody (1:10,000) and goat anti-rabbit IgG (H + L) cross-adsorbed secondary antibody (1:10,000), Alexa Fluor™ 594 (Thermo Fisher Scientific).

### cDNA synthesis and semi-quantitative RT-PCR

Ovaries were dissected from *vit* mutant, *vit/+*, *dazao,* and *BmVgR + vit* strains during the moth stage; the samples were flash-frozen in liquid nitrogen and stored at −80°C. Total RNA was extracted using the TRIzol Reagent kit (Life Technologies, United States); Moloney Murine Leukemia Virus Reverse Transcriptase (M-MLV RT; Promega, United States) was used to generate first-strand cDNA. All assays were done according to manufacturers’ instructions.

Unlike *dazao*, the *vit* mutant had abnormal BmVgR, with a 228-bp deletion in the third-class B region of the first EGF domain (Lin et al., 2013). Primers were designed based on the *vit*-deleted gene fragment (abbreviated as *vit’*). The forward primer (*vit’*-F) was 5ʹ-CTC​CAT​ATG​TAC​CAC​CCA​GCG​TTG​A-3ʹ, and the reverse primer (*vit’*-R) was 5ʹ-GGG​GAT​GCA​TCT​GCC​GTT​CTT​GTT​T-3ʹ. The PCR conditions were 94°C for 5 min, followed by 27 cycles of 94°C for 30 s, 62°C for 30 s, and 72°C for 15 s, and a final extension at 72°C for 10 min *Bmactin3* was used as an internal reference; the forward primer was 5ʹ-AAC​ACC​CCG​TCC​TGC​TCA​CTG-3ʹ, and the reverse primer was 5ʹ-GGG​CGA​GAC​GTG​TGA​TTT​CCT-3ʹ. PCR was performed at 94°C for 5 min, followed by 25 cycles of 94°C for 30 s, 53°C for 30 s, and 72 °C for 30 s, and a final extension at 72°C for 10 min. The *vit’* and *Bmactin3* cDNA fragments were PCR-amplified using rTaq DNA polymerase (Takara, Otsu, Japan). The PCR products were confirmed by electrophoresis on 1.2% (w/v) agarose gel and photographed using the Molecular Imager Gel Doc XR system (Bio-Rad, United States).

## Results

### Sequence analysis of the 30 K proteins

**FIGURE 1:**
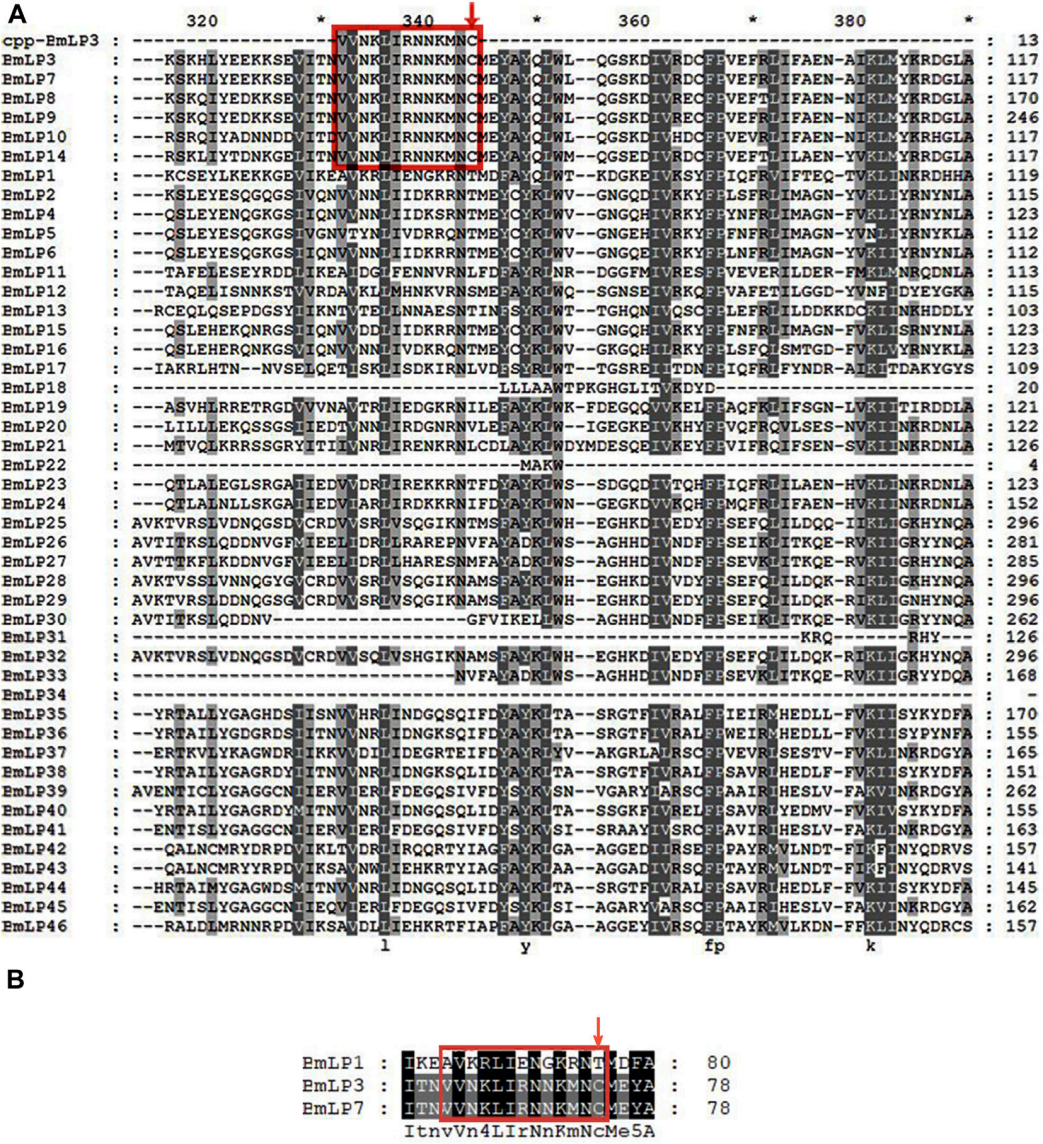
Sequence analysis of 30 K proteins in silkworm. **(A)** Alignment of 46 types of silkworms 30 K proteins using Clustalx1.83 and GENEDOC. **(B)** Alignment of partial amino acid residues of BmLP1, BmLP3, and BmLP7 using Clustalx1.83 and GENEDOC. 30 K protein sequences were downloaded from the Silkworm Genome Database (http://silkdb.bioinfotoolkits.net/). Residues in the red box represent the common CPP domain in partial 30 K proteins, including BmLP3 and BmLP7. The red down arrow indicates the conserved domain of 30 K proteins consistent with the Cysteine position of the CPP domain of BmLP3.

BmLP1, BmLP3, and BmLP7 belong to the 30 K family. We compared the sequence of CPP-BmLP3 with 46 types of 30 K proteins in the silkworms. The results indicated that BmLP7, BmLP8, BmLP9, BmLP10, BmLP14, besides BmLP3 (amino acid residues marked with red boxes) contained the CPP domain and Cysteine position (Figure 1A). However, 40 types of 30 K proteins, including BmLP1, were devoid of the CPP domain and Cysteine position (Figure 1B). These results suggest that BmLP7, a 30 K protein with the CPP domain and Cysteine position, might be related to BmLP3 (Park et al., 2014a; Park et al., 2014b), which gains entry into oocytes without the aid of receptors. However, 30 K proteins such as BmLP1, which are without a CPP domain and Cysteine position, might be transported into oocytes by receptors.

### BmLP1 without CPP domain and cysteine position could not directly enter sf9 cells

**FIGURE 2:**
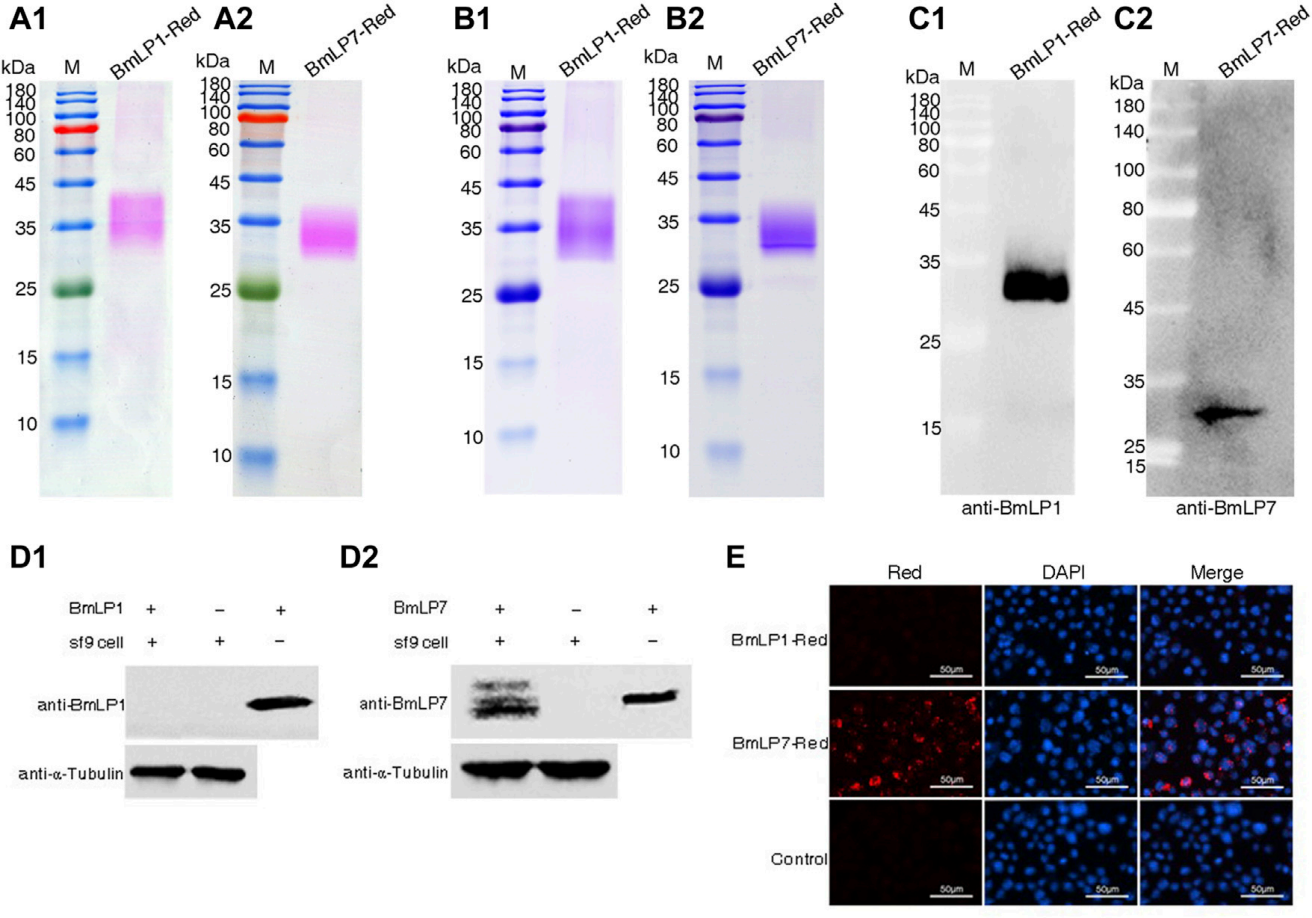
Analysis of BmLP1 and BmLP7 entering sf9 cells. **(A)** Direct observation of BmLP1-Red (A1) and BmLP7-Red (A2) after SDS-PAGE. **(B)** Coomassie brilliant blue staining detection of the BmLP1-Red (B1) and BmLP7-Red (B2) after SDS-PAGE. **(C)** Detection of BmLP1-Red (C1) and BmLP7-Red (C2) using western blotting by anti-BmLP1 and BmLP7 rabbit antibody (1:10,000), respectively, and HRP-conjugated goat anti-rabbit IgG (H + L) antibody (1:10,000). **(D)** Detection of BmLP1 (D1) and BmLP7 (D2) proteins entering sf9 cells. Lane 1, sf9 cells lysate after incubation with BmLP1 or BmLP7; Lane 2, sf9 cells lysate without BmLP1 or BmLP7 protein; Lane 3, purified BmLP1 or BmLP7 protein; α-tubulin, used as an internal reference. **(E)** Fluorescence signal detection of BmLP1-Red and BmLP7-Red entering sf9 cells. Red images indicate cells observed under the red fluorescence-emitting light channel. DAPI images indicate cells observed with DAPI nuclear counter stain. Merged images show a combination of Red and DAPI images. Control, sf9 cells incubated with PBS.

To analyze whether BmLP1 (without CPP domain and Cysteine position) and BmLP7 (with CPP domain and Cysteine position) could enter into the sf9 cells without BmVgR, we used pHrodo-Red dye to label the BmLP1 and BmLP7 proteins, which were purified from the hemolymph at day two of silkworm pupation. SDS-PAGE indicated rose-red bands under white light, which represented BmLP1-Red (Figure 2A1) and BmLP7-Red proteins (Figure 2A2); there were blue bands at the same location after staining with Coomassie brilliant blue (Figure 2B1B2). These locations of protein bands were further confirmed using western blotting, which detected BmLP1 (Figure 2C1) and BmLP7 (Figure 2C2) at the same sites. These results indicated that the BmLP1 and BmLP7 proteins were successfully labeled by pHrodo-Red. Western blotting analysis of sf9 cells incubated with 1 mg/mL BmLP1-Red or BmLP7-Red showed that BmLP1 could not directly enter into the sf9 cells (Figure 2D1), whereas BmLP7 was detected in the sf9 cells (Figure 2D2). This was further confirmed by fluorescence microscopy; the fluorescence signal of BmLP1-Red was not detected in the sf9 cells, while that of BmLP7-Red was detected in the sf9 cells (Figure 2E). These results suggest that BmLP1 might enter into the cells with the help of receptors, whereas BmLP7 could directly enter into sf9 cells without receptors.

### Analysis of BmVgR binding and transporting BmLP1

**FIGURE 3:**
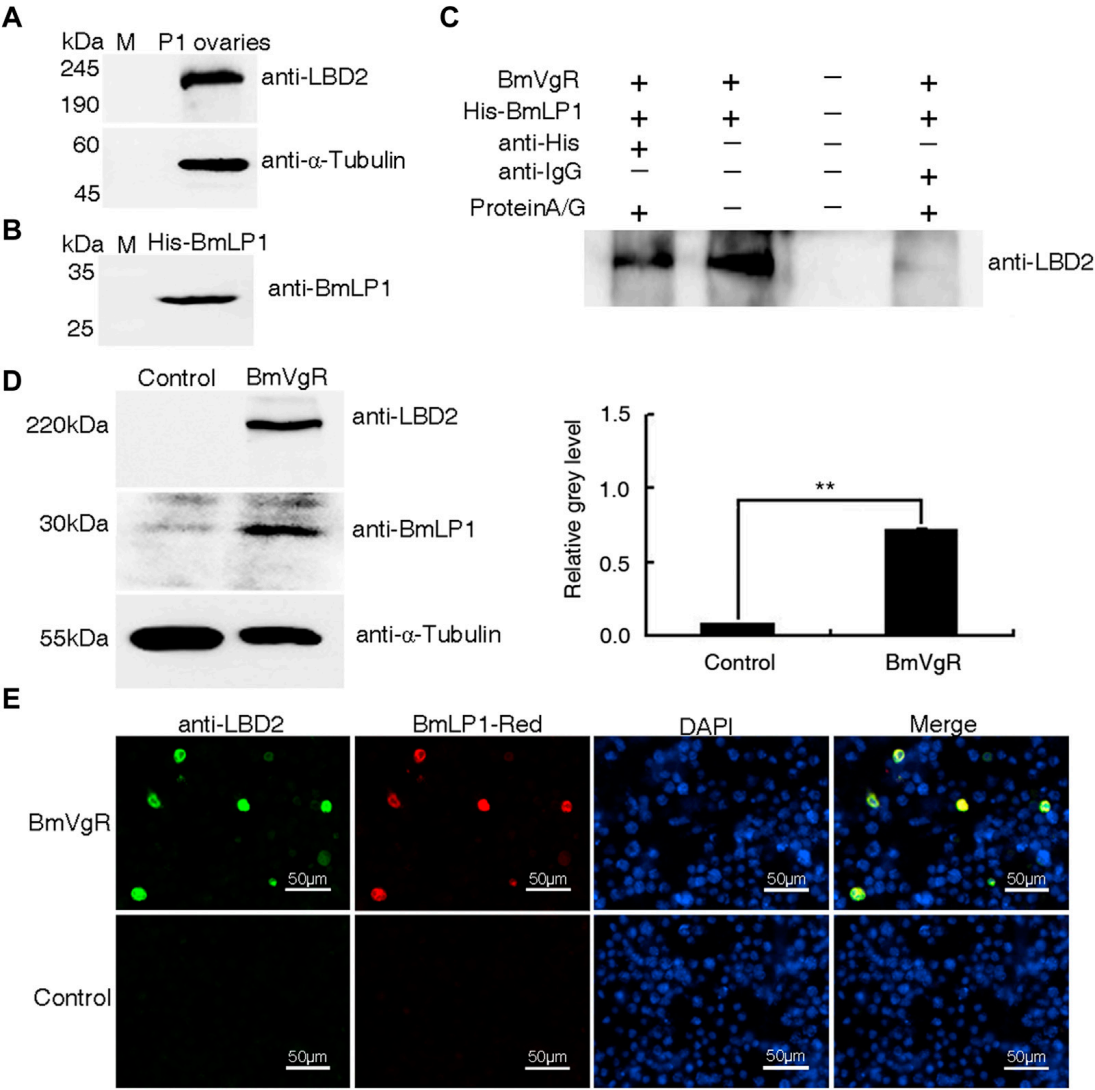
Binding and transport of BmLP1 by BmVgR into the sf9 cells. **(A)** Western blot detection of BmVgR proteins from silkworm ovaries on day one of pupation. **(B)** Detection of His-BmLP1 by western blotting. **(C)** Detection of BmVgR interaction with His-BmLP1 by Co-IP. Lane1, elution from the anti-His beads (sample); Lane2, input BmVgR and His-BmLP1 (positive control); Lane3, last washing buffer for Co-IP (negative control); Lane 4, elution from the anti-IgG beads (negative control). **(D)** Detection of BmLP1 proteins transported into sf9 cells over-expressing BmVgR. On the left is the results of western blotting; to the right is the grayscale analysis of protein bands in western blotting using ImageJ. Experiments were repeated three times independently; the relative gray-level is shown as the mean ± SD; **, *p* < 0.01. **(E)** Fluorescence detection of BmLP1 proteins transported into sf9 cells. Anti-LBD2 images indicate the localization of BmVgR (Green); BmLP1-Red images indicate the localization of BmLP1 (Red); DAPI represents the localization of the cell nucleus (Blue); Merged images show the combination of anti-LBD2, BmLP1-Red, and DAPI images. Control: sf9 cells. α-Tubulin was used as the internal reference. Samples were detected using anti-LBD2 rabbit antibody (1:10,000), anti-BmLP1 rabbit antibody (1:10,000), HRP-conjugated goat anti-rabbit IgG (H + L) antibody (1:10,000), or goat anti-rabbit IgG (H + L) cross-adsorbed secondary antibody (1:10,000)- Alexa Fluor™ 488.

To analyze whether BmLP1 is transported by BmVgR, we performed an *in vitro* assay to determine the interaction between BmVgR and BmLP1. Western blotting analysis revealed the presence of BmVgR in the ovaries of the silkworm *dazao* on day one of pupation (Figure 3A). BmLP1 (His-BmLP1) purified *via* prokaryotic expression was detected by western blotting (Figure 3B). Co-IP confirmed the binding of BmVgR with His-BmLP1 (Figure 3C).

For assays, we selected sf9 cells that did not express endogenous *BmVgR*. After incubation with BmLP1-Red, western blotting and grayscale analysis showed that the amount of BmLP1 increased in sf9 cells, which over-expressed BmVgR, compared to that in cells not over-expressing BmVgR (Figure 3D). Additionally, the fluorescence signal was also clearly observed in the sf9 cells. Both BmVgR and BmLP1-Red were co-localized in the same sf9 cells under fluorescence microscope (Figure 3E). These results indicate that BmVgR mediates the transport of BmLP1 into sf9 cells.

### Expression of truncated BmVgR (SE11C and SE22C) in sf9 cells

**FIGURE 4:**
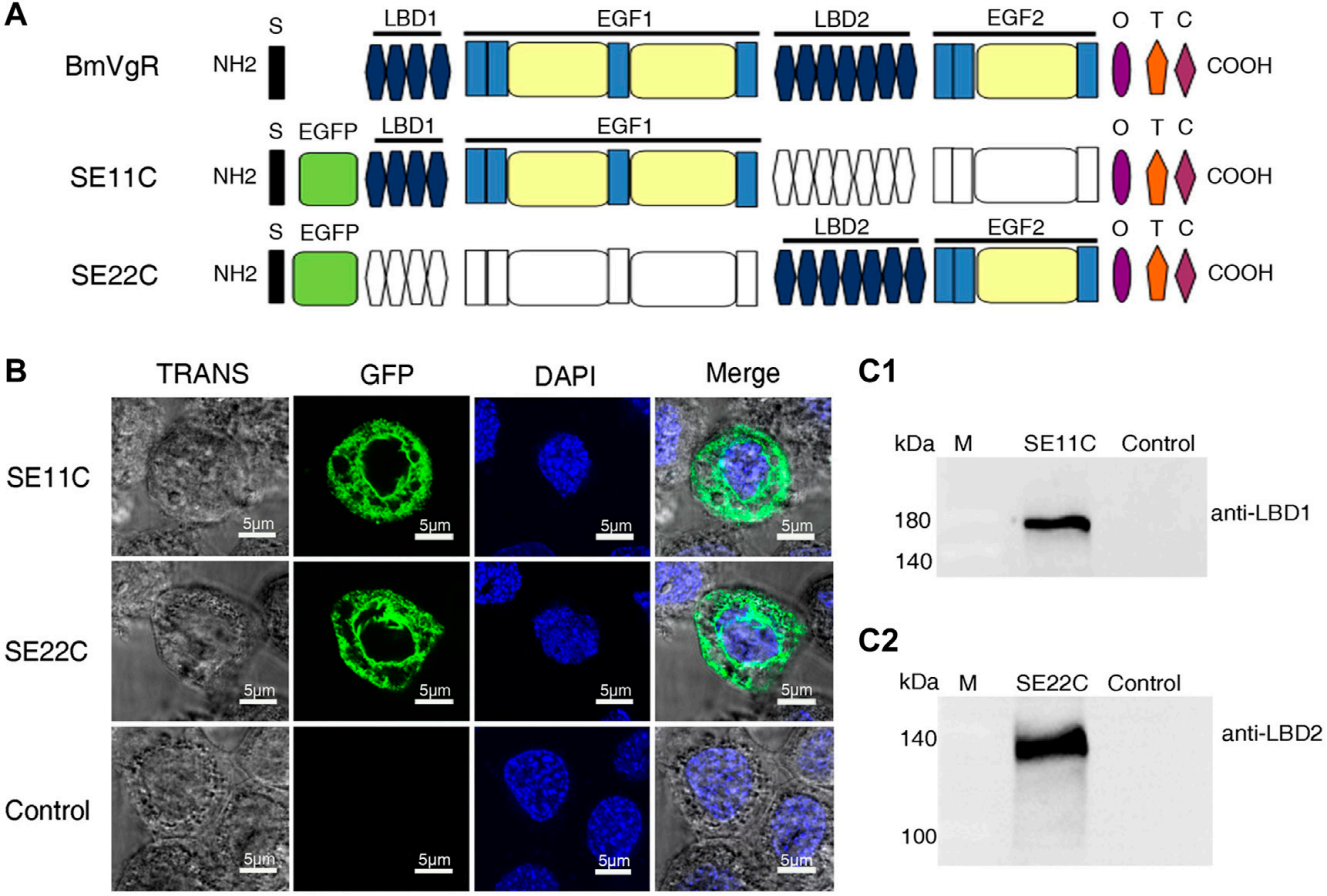
Structure and localization of SE11C and SE22C. **(A)** Structural schematic diagram of BmVgR, SE11C, and SE22C. S, signal peptide; EGFP, enhanced green fluorescence protein tag; LBD, ligand-binding domain; EGF, epidermal growth factor precursor homology domain; O, O-linked sugar domain; T, transmembrane domain; C, cytoplasmic domain. The colorless box indicates the missing corresponding domains; the color box indicates that it constitutes the corresponding domains. **(B)** Detection of SE11C and SE22C in sf9 cells using green fluorescence under a laser confocal microscope. Sf9 cells over-expressing SE11C and SE22C, respectively; Control: sf9 cells. TRANS images show the bright field image of cells; GFP images indicate the localization of SE11C and SE22C (Green); DAPI images show the nuclear counterstain (Blue); The Merge images combine the images of TRANS, GFP, and DAPI. **(C)** Expression of SE11C (C1) and SE22C (C2) in sf9 cells detected by western blotting. Samples were detected using western blotting with anti-LBD1 or anti-LBD2 rabbit antibody (1:10,000) and HRP-conjugated goat anti-rabbit IgG (H + L) antibody (1:10,000).

To further illustrate whether the additional LBD1+EGF1+OTC domain (SE11C) and the LBD2+EGF2+OTC domain (SE22C) of BmVgR could transport BmLP1, we constructed truncated cell expression vectors with a SE11C or SE22C structure (Figure 4A). Fluorescence observation showed that both SE11C and SE22C could be stably expressed in sf9 cells; SE11C and SE22C were localized in the cell membrane and cytoplasm (Figure 4B). Western blotting showed that the molecular weight of SE11C and SE22C proteins was approximately 180 kDa (Figure 4C1) and 140 kDa (Figure 4C2), respectively. The results revealed that the truncated BmVgR vectors were successfully expressed in sf9 cells.

### Analysis of SE11C and SE22C binding and transporting BmLP1

**FIGURE 5:**
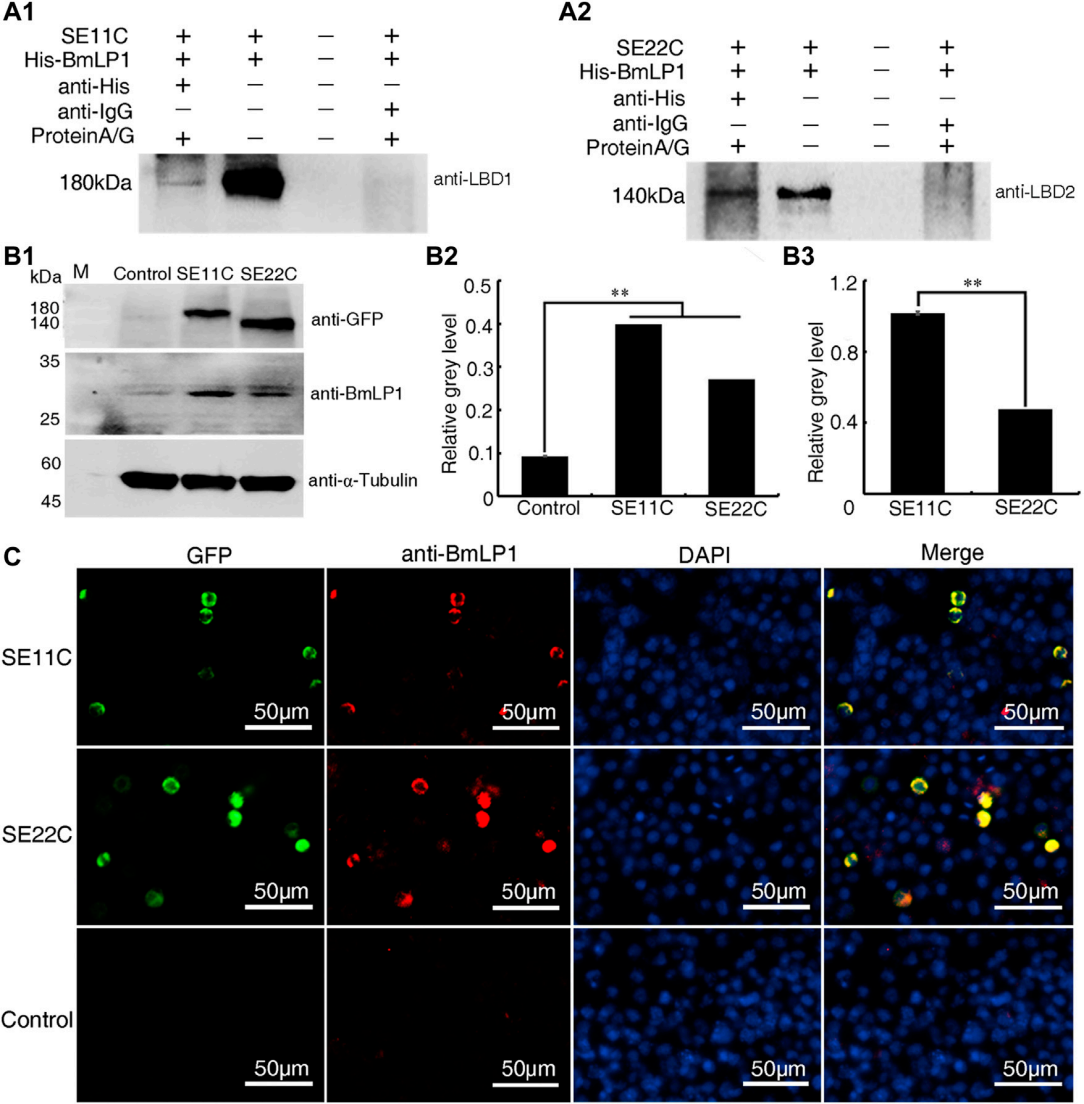
Analysis of SE11C and SE22C binding with the BmLP1 *in vitro* and mediating transport into sf9 cells. **(A)** Analysis of SE11C (A1) and SE22C (A2) binding with BmLP1 by Co-IP. Samples were subjected to SDS–PAGE (10% w/v polyacrylamide gels) and detected using western blotting by anti-LBD1/anti-LBD2 rabbit antibody (1:10,000), respectively, and HRP-conjugated goat anti-rabbit IgG (H + L) antibody (1:10,000). Lane1, elution from anti-His beads (sample); Lane2, input SE11C/SE22C and His-BmLP1 proteins (positive control); Lane3, last washing buffer for Co-IP assay (negative control); Lane 4, elution from anti-IgG beads (negative control). **(B)** Detection of the BmLP1 proteins transported into sf9 cells over-expressing SE11C or SE22C. (B1) Results of western blotting. Proteins were detected using anti-GFP (1:10,000), anti-BmLP1 (1:10,000) polyclonal rabbit antibody, and HRP-conjugated goat anti-rabbit IgG (H + L) antibody (1:10,000). Grayscale analysis of the protein bands in western blotting using ImageJ. α-Tubulin was used as the internal reference (B2) SE11C or SE22C protein over-expressed as an internal reference (B3). Experiments were repeated three times independently, and the relative gray level was shown as mean ± SD; **, *p* < 0.01. **(C)** Fluorescence detection of BmLP1 proteins transported into the sf9 cells by fluorescence microscope (Life Technologies, USA). Detection used anti-BmLP1 (1:10,000) and Goat anti-Rabbit IgG (H + L) Cross-Adsorbed Secondary Antibody, Alexa Fluor™ 594. GFP images indicate the localization of SE11C or SE22C (Green); anti-BmLP1 images indicate the localization of BmLP1 protein (Red); DAPI images are shown with nuclear counter stain (Blue); Merged images indicate a combination of GFP, anti-BmLP1, and DAPI images. Control: sf9 cells.

To confirm the binding of SE11C and SE22C with BmLP1, His-BmLP1 was incubated with the lysate of sf9 cells with SE11C and SE22C over-expression, respectively. Co-IP showed that both SE11C (Figure 5A1) and SE22C (Figure 5A2) could bind to BmLP1.

Further, we investigated whether SE11C and SE22C could transport BmLP1 into sf9 cells. We incubated SE11C or SE22C over-expressed sf9 cells with 1 mg/mL BmLP1. We performed western blotting to detect BmLP1 in sf9 cell lysates. The results showed that BmLP1 was transported into the sf9 cells over-expressing SE11C or SE22C (Figure 5B1). Through the grayscale analysis the bands of the western blotting, the amount of BmLP1 was significantly increased after the over-expression of SE11C or SE22C in sf9 cells when α-tubulin was used as the internal reference (Figure 5B2). When SE11C and SE22C proteins were used as the internal reference, SE11C might showed a stronger ability to transport BmLP1 than SE22C (Figure 5B3). Immunofluorescence assays showed that SE11C and SE22C shared a common fluorescence localization with BmLP1 in the sf9 cells (Figure 5C). These findings further confirmed that truncated BmVgR is able to transport BmLP1 into the sf9 cells.

### Detection of BmLP1 in the hemolymph and ovaries of the BmVgR mutant

**FIGURE 6:**
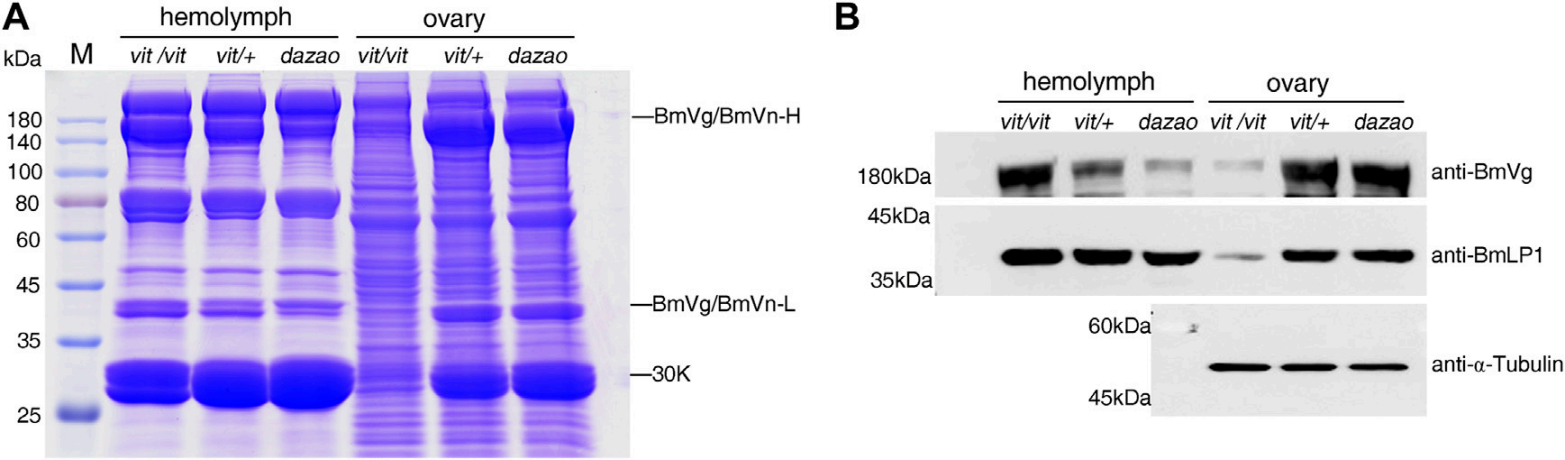
Detection of BmLP1 and BmVg/BmVn in *BmVgR* mutant hemolymph and ovaries. **(A)** Detection of total proteins from silkworm hemolymph and ovaries of the *vit mutant*, *vit/+*, and the *dazao* strains on day five of pupation by SDS-PAGE (25 μg/lane). **(B)** Detection of the BmVg/BmVn and BmLP1 proteins in the hemolymph and ovaries of *vit mutant*, *vit/+*, and *dazao* by western blotting (30 μg/lane) on day five of pupation. *Vit/+* and *dazao* were used as controls. Anti-BmVg and anti-BmLP1 rabbit antibodies and HRP-conjugated goat anti-rabbit IgG (H + L) antibody were used at a dilution of 1:10,000.

The phenotype of the *vit* mutant was due to BmVgR not normally transporting ligands into oocytes, as exhibited by BmVg and 30 K proteins (Lin et al., 2013). This study compared the total protein of the hemolymph and ovaries of the *vit* mutant, *vit/+*, and *dazao* on day five of pupation. SDS-PAGE and western blotting analyses indicated that the *vit* mutant lacked BmVn and 30 K proteins including BmLP1 in the ovaries; however, BmVg and 30 K proteins including BmLP1 were abundant in the hemolymph. Contrarily, low amounts of BmVg and 30 K proteins including BmLP1 were detected in the hemolymph, while higher amounts were detected in the eggs of *vit/+* and *dazao*. (Figures 6A,B). These results indicated that mutant BmVgR affected not only the transport of BmVg but also that of BmLP1 into the ovaries.

### Functional rescue of BmVgR-mediated transport of BmLP1 in *vit* mutants by transgene

**FIGURE 7:**
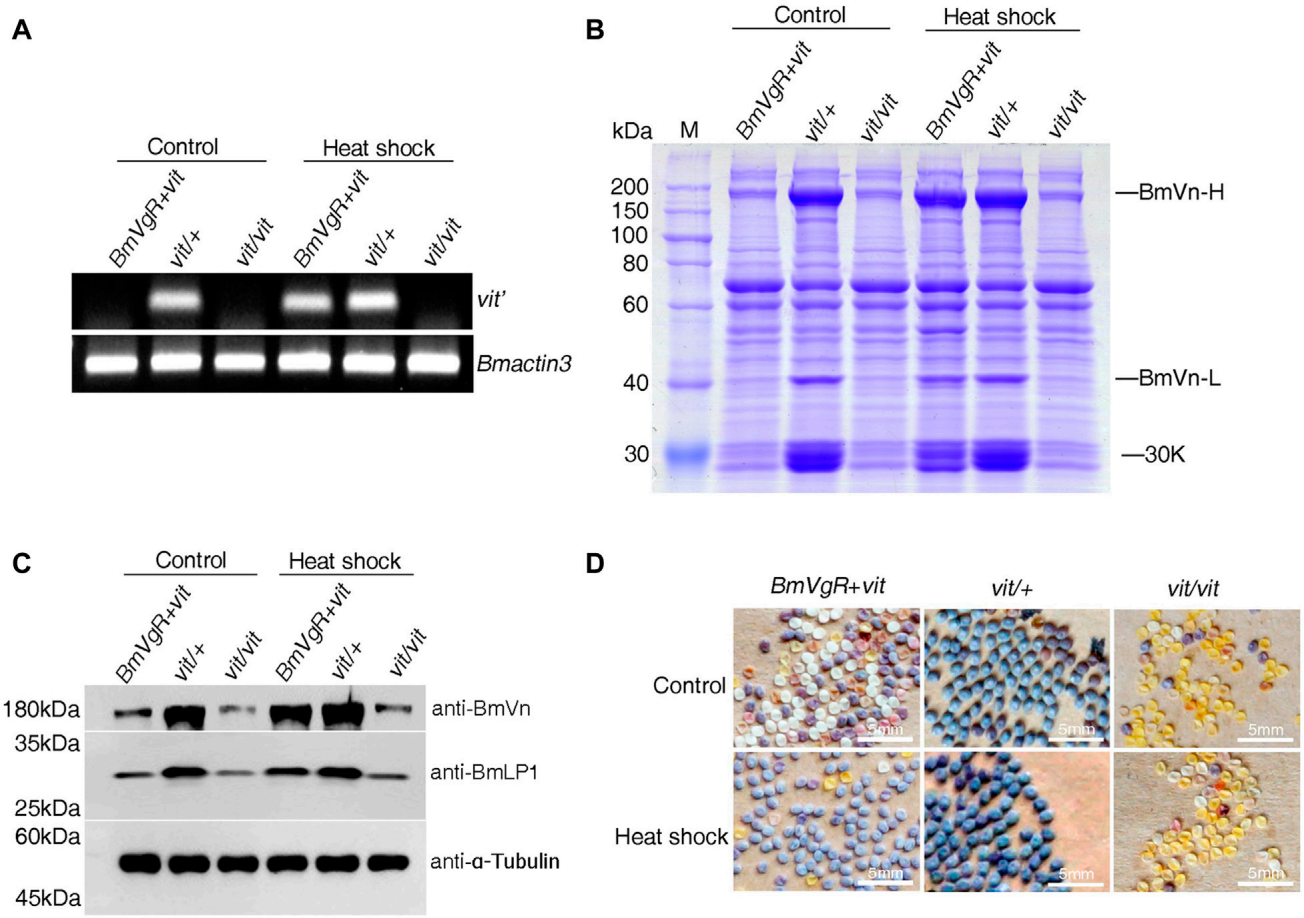
*BmVgR* expression and phenotype of the transgenic *BmVgR + vit* after heat shock. **(A)** *Vit’* gene fragment in the ovaries of the *BmVgR + vit* after heat shock by RT-PCR; *Bmactin3* is used as an internal reference. **(B)** The detection of total proteins in the ovaries of the *BmVgR + vit* after heat shock treatment by using SDS-PAGE. **(C)** Detection of BmVn and BmLP1 proteins in the ovaries of the *BmVgR + vit* after heat shock treatment by using western blotting. α-Tubulin was used as an internal reference. Anti-BmVg, anti-BmLP1 rabbit, and HRP-conjugated goat anti-rabbit IgG (H + L) antibodies were used at a dilution of 1:10,000. **(D)** The phenotype of silkworm eggs in the offsprings of the *BmVgR + vit* after heat shock. *Vit/+* was used as a control. Heat shock, treatment at 27°C, 32°C, 37°C, and 42°C for 1 h on days 1, 3, 5, and 7 of pupation, respectively. Control, treatment at 27 °C from corresponding stages of pupation like samples.

To further confirm whether BmVgR is able to transport BmLP1 into silkworm oocytes, we selected the transgenic strain of *vit* mutant with hsp70 promoter (*BmVgR* + *vit*); *vit/+* was used as a control. Twenty female pupae from each strain were treated at 27°C, 32°C, 37°C, and 42°C for 1 h on 1, 3, 5, and 7 days of pupation, respectively. Control strains were treated at 27 °C from corresponding stages of pupation with sample strains. After the heat shock treatment, we checked the presence of deleted fragments of the *vit* mutant (*vit’*) in the ovaries of the heat shock-treated and control strains by RT-PCR. The results showed that *vit’* was detected in the ovaries of *BmVgR + vit*, as well as those of the control post heat shock-treatment. However, the *vit’* of the *BmVgR + vit* was not detected like that of the *vit* mutant with no heat shock treatment (Figure 7A). SDS-PAGE analysis of total protein from control and treated female moth ovaries indicated an increase in the BmVn and 30 K proteins in *BmVgR + vit* ovaries than those in the *vit* mutant ovaries after heat shock treatment (Figure 7B). Similar results were obtained with western blotting; there was an increased expression of BmVn and BmLP1 proteins in the *BmVgR + vit* ovaries than that detected in the *vit* mutant ovaries after the heat shock treatment (Figure 7C).

Additionally, we selected six female moths of *BmVgR + vit* that had mated with male *vit* mutant moths and observed the phenotypes of the eggs. *BmVgR + vit* laid more purple and plump eggs after the heat shock treatment compared to the small, white, and shriveled eggs of the *vit* mutant (Figure 7D). These results showed that the hsp70 promoter was induced by the heat shock treatment, which in turn activated the expression of *BmVgR* in *BmVgR + vit*, and partly rescued the function of mutated BmVgR in the *vit* mutant. The results further confirmed the function of BmVgR, which transports BmVg and 30 K proteins such as BmLP1 into oocytes and promotes the formation and development of silkworm eggs.

## Discussion

Like BmVn, 30 K proteins are important components of egg yolk proteins in silkworm eggs, providing nutrients for egg formation and later embryonic development. Park et al. discovered that BmLP3, one of the 30 K proteins with a CPP and Cysteine position, could efficiently penetrate cells grown in a medium used for mammalian cell culture (Park et al., 2014a). However, whether the other 30 K proteins require receptor-mediated transport is unknown. In this study, we compared the sequence of 46 reported 30 K proteins and found CPP domain and Cysteine position in five proteins *viz.* BmLP7, BmLP8, BmLP9, BmLP10, and BmLP14, similar to that of BmLP3. This suggests that most of the 30 K proteins might enter silkworm oocytes using receptors. We randomly selected BmLP1 (without CPP domain and Cysteine position) and BmLP7 (with CPP domain and Cysteine position) for our experiments. Our results indicated that 30 K proteins with CPP domain and Cysteine position, such as BmLP3/BmLP7, could gain direct entry into the cells independent of the receptors. The transportation of 30 K proteins, such as BmLP1, which do not contain the CPP domain and Cysteine position, might be mediated by receptors.

Co-IP of total proteins from the ovaries incubated with the His-BmLP1 obtained from prokaryotic expression showed that BmVgR could bind with His-BmLP1. BmLP1-Red, when incubated with sf9 cells over-expressing BmVgR, showed that BmLP1-Red proteins could be transported into the sf9 cells over-expressing BmVgR. We found that the additional LBD1+EGF1+OTC (SE11C) and LBD2+EGF2+OTC (SE22C) domains could also transport BmLP1 into the cells; the ability of SE11C to transport BmLP1 might be stronger than that of SE22C. The results obtained using western blotting, Co-IP, and fluorescence imaging revealed that BmVgR with different LBDs could transport multiple ligands. These results correspond to those of previous studies which demonstrated that VgRs recognize multiple ligands (Sappington et al., 1996; Tufail and Takeda, 2007; Shu et al., 2011; Smith and Reuben Kaufman, 2013; Zhong et al., 2015). LRP recognizes at least 30 different ligands (Herz and Strickland, 2001); chicken VgR recognizes at least eight different ligands (Jacobsen et al., 1995). Different repeat regions of the LBRs have different affinities to ligands (Esser et al., 1988; Li et al., 2003). Among them, LBD is mainly involved in the interaction between receptors and ligands, while EGF plays a key role in the dissociation of receptors and ligands under acidic conditions. The phosphorylation or dephosphorylation of the EGF domain will also affect the binding and dissociation of receptors and ligands (Jing et al., 2021). Compared with VgRs of other species, insect VgRs have two LBD + EGF domains. Whether the additional domain and the original domain differ in the number and type of transport of ligands are rarely reported. Although LBR sequences are highly conserved, their capacity to transport ligands might differ. The binding ability of LBRs to different ligands is predominantly determined by the number and arrangement of LBRs in LBDs (Jacobsen et al., 1995; Kounnas et al., 1996; Li et al., 2003; Liu et al., 2016). In silkworms, BmVg and 30 K proteins constitute 40% and 35% of the total silkworm yolk protein, respectively. There are 40 types of 30 K proteins, including BmLP1, which do not contain the CPP domain and Cysteine position and may require more powerful receptors for transport. This suggests that the function of the additional LBD1+EGF1 might transport more ligands.

We used the *BmVgR* mutant as the experimental material for individual biological verification. The *vit* mutant represents the abnormal function of BmVgR owing to the lack of BmVn and 30 K proteins in the ovary (Lin et al., 2013). We used *BmVgR* + *vit* transgenic strain with hsp70 promoter as the experimental material. After heat treatment at different pupal stages, the change in egg phenotype of BmVgR + *vit*, as well as the expression of *BmVgR*, in addition to the increased accumulation of BmVn and BmLP1 in the *BmVgR + vit* ovaries indicated that the transport function of *BmVgR + vit* is rescued to some extent by the heat shock treatment. These results further confirmed that BmVgR is capable of transporting BmLP1 besides BmVg. It has been reported in white perch (*Morone americana*) that multiple forms of VtgR bind to multiple types of Vtg (Reading et al., 2011). The LBD1 of BmVgR consists of four LBRs, and the LBD2 of BmVgR consists of seven LBRs. Multiple forms of BmVgR are expressed throughout the lifespan of silkworms (data not shown), which may bind to other non-CPP 30 K protein ligands besides BmLP1 and BmVg. In *Oreochromis aureus*, Lys185, a positively charged residue, plays a crucial role in the receptor binding of Vtg (Li et al., 2003). Yet, the key amino acid residue for the binding of BmLP1 to BmVgR remains unidentified. The molecular mechanisms need to be explored in future studies.

CPPs and their protein transduction domains have been used to deliver drugs and proteins into the cells *via* receptor-independent endocytosis (Park et al., 2012). BmLP3/BmLP7 can be used as drug delivery tools to deliver cargo molecules, including proteins, to the target organs or tissues; these proteins could be applied in treating diseases such as cancer in mammals and humans. Pathogenic microbes are known to bind to a secreted ligand protein, hitchhiking the ligand-receptor pathway to achieve cell entry (Huo et al., 2019; Mitchell et al., 2019). In the small brown plant hopper, the vitellogenin receptor plays an essential role in the vertical transmission of the rice stripe virus during oogenesis (He et al., 2019). Since silkworm is a lepidopteran model insect, deciphering the mechanisms of BmVgR transport ligands in silkworms would help in the ecological control of lepidopteran pests and may provide new insights for disease control in silkworms.

## Data Availability

The original contributions presented in the study are included in the article/supplementary material, further inquiries can be directed to the corresponding author/s.
